# SEOM clinical guidelines for the treatment of metastatic prostate cancer (2017)

**DOI:** 10.1007/s12094-017-1783-2

**Published:** 2017-11-13

**Authors:** J. Cassinello, J. Á. Arranz, J. M. Piulats, A. Sánchez, B. Pérez-Valderrama, B. Mellado, M. Á. Climent, D. Olmos, J. Carles, M. Lázaro

**Affiliations:** 1 Medical Oncology Department, Hospital Universitario de Guadalajara, Guadalajara, Spain; 2 Medical Oncology Department, Hospital Universitario Gregorio Marañón, Madrid, Spain; 3 Medical Oncology Department, Institut Català d’Oncologia, L’Hospitalet de Llobregat, Barcelona, Spain; 4 Medical Oncology Department, Consorcio Hospitalario Provincial de Castellón, Castellón de la Plana, Spain; 5 Medical Oncology Department, Hospital Universitario Virgen del Rocío, Sevilla, Spain; 6 Medical Oncology Department, IDIBAPS, Hospital Clinic, Barcelona, Spain; 7 Medical Oncology Department, Fundacion Instituto Valenciano de Oncología, Valencia, Spain; 8 CNIO-IBIMA Genito-Urinary Cancer Unit, Medical Oncology Department, Hospitales Universitarios Virgen de la Victoria y Regional de Málaga, Malaga, Spain; 9 Vall d’Hebron University Hospital, Vall d’Hebron Institute of Oncology, Barcelona, Spain; 10 Medical Oncology Department, Hospital Universitario de Vigo, Vigo, Spain

**Keywords:** Androgen deprivation treatment, Hormone-sensitive advanced prostate cancer, Castration-resistant prostate cancer, Abiraterone, Enzalutamide, Radium 223, Cabazitaxel, Docetaxel

## Abstract

Androgen deprivation treatment was the only treatment available for metastatic prostate cancer until recently, with docetaxel as the only treatment with a proven survival benefit in castration-resistant prostate cancer (CRPC). Several drugs have been approved in the castration-resistant disease (sipuleucel-T, cabazitaxel, abiraterone, enzalutamide, radium-223). More recently, docetaxel and abiraterone have been moved to the hormone-sensitive disease setting, achieving better patient survival. The purpose of this article is to define the state of the art in the treatment of prostate carcinoma.

## Introduction

According to the International Agency for Research on Cancer, prostate cancer was the most incident and prevalent malignancy in Spain in 2012. There were 27,853 new prostate cases diagnosed in Spain and 399,964 in Europe during this period, with an age-standardized rate of incidence and mortality per 100,000 inhabitants of 96.8 and 15.2 in Spain, and 92.1 and 19.3 in Europe, respectively. Predictions show that about 33,000 and 455,732 new cases will be detected in Spain and Europe, respectively, in 2020 [1, 2]. Approximately 30% of prostate cancer patients will develop advanced disease, and eventually all of them will develop a progressive disease, a status called castration-resistant prostate carcinoma (CRPC).

Medical oncologists should be aware that in the last few years important advances have been made in the treatment of metastatic prostate cancer, particularly in the metastatic setting, where new drugs and new therapeutic strategies have emerged associated with a clear survival benefit. On the other hand, these new therapies are associated with an increase in financial costs as well as with potential adverse effects, particularly in the more aged population of patients with prostate cancer.

In this context, the Spanish Society of Medical Oncology (SEOM), in collaboration with the Spanish Genitourinary Oncology Group (SOGUG), has decided to review in this guide the current state of knowledge in the treatment of metastatic prostate cancer, as in 2014, when first guide was published [3]. All the authors reviewed the literature available until May 2017. After a synthesis and discussion, the present document was written which synthesizes the current state of knowledge and offers evidence-based recommendations. The aim of this work is to offer a practical guide of recommendations for the oncologist involved in the management of metastatic prostate cancer that facilitate decision-making, allowing the patient to be offered the best therapeutic available option, and considering at the same time the best use of resources and the minimization of the side effects associated with the treatment.

## Methodology

The SEOM guidelines have been developed with the consensus of ten genitourinary cancer oncologists from SEOM and SOGUG.

To assign a level of levels of evidence and grades of recommendation, we have used Table 1 [4].

**Table 1:** Levels of evidence/grades of recommendation

Levels of evidence
I: Evidence from at least one large randomised, controlled trial of good methodological quality (low potential for bias) or meta-analyses of well-conducted randomised trials without heterogeneity
II: Small randomised trials or large randomised trials with a suspicion of bias (lower methodological quality) or meta-analyses of such trials or of trials with demonstrated heterogeneity
III: Prospective cohort studies
IV: Retrospective cohort studies or case–control studies
V: Studies without control group, case reports, experts opinions
Grades of recommendation
A: Strong evidence for efficacy with a substantial clinical benefit, strongly recommended
B: Strong or moderate evidence for efficacy but with a limited clinical benefit, generally recommended
C: Insufficient evidence for efficacy or benefit does not outweigh the risk or the disadvantages; optional
D: Moderate evidence against efficacy or for adverse outcome, generally not recommended
E: Strong evidence against efficacy or for adverse outcome, never recommended

Statements without grading were considered justified standard clinical practice by the SEOM and SOGUG faculty and experts.

In algorithms 1 and 2, we summarize our recommendations.

## Hormone-sensitive disease

### Androgen deprivation therapy (ADT)

It can be achieved suppressing the secretion of testicular androgens or inhibiting the action of circulating androgens with compounds known as anti-androgens. Surgical castration is still considered the ‘gold standard’ for ADT and the standard castrate level is < 50 ng/mL (1.7 nmol/L). Nevertheless, current testing methods have found that the mean value after surgical castration is 15 ng/mL. LHRH agonist are currently the main form of ADT. They are delivered as depot injections on a 1-, 2-, 3-, or 6-month basis. After the first injection, they induce a transient rise in LH and FSH leading to a testosterone surge and flare-up phenomenon that can clinically be avoid adding an antiandrogen for the first month of therapy. Luteinizing hormone-releasing hormone (LHRH) antagonists bind immediately to LHRH receptors of pituitary gland, with a rapid decrease of testosterone level without any flare. Any of them (surgical castration, LHRH agonist or antagonist) are standard of care. Antiandrogens compete with androgens at the receptor level and they are classified according to their chemical structure as steroidal (cyproterone acetate, megestrol acetate, medroxyprogesterone acetate) and non-steroidal (nilutamide, flutamide, bicalutamide) [5].

Complete androgen blockade (CAB) refers to the combination of a LHRH agonist and an antiandrogen. Systematic reviews show that CAB using a nonsteroidal antiandrogen provides only a small survival advantage (< 5% at 5 years) versus monotherapy, but increasing side effects and economic costs.

### PSA recurrence, M0 disease

Despite primary tumor curative treatments as radical prostatectomy (RP) or radical radiotherapy (RT), up to 25–50% of patients will develop PSA recurrence. Following RP, recurrent cancer may be defined by two consecutive PSA values of > 0.2 ng/mL and rising. After primary RT, the Phoenix Consensus defines as an increase > 2 ng/mL higher than the PSA nadir value, regardless of the serum concentration of the nadir. The natural history of PSA-only rising patient can be very long, so treatment recommendations should be given after discussion inside a multidisciplinary team: we must try to delay metastatic disease and death, but we also must avoid over-treatment, as many patient’s disease will neither affect their survival nor quality of life.

Risk factors for metastases in a patient with PSA recurrence following RP are: doubling-time (PSA-DT) < 3 months, seminal vesicle invasion, Gleason score 8–10, or time to PSA recurrence < 3 years. On the other hand, a very low-risk can be defined as patients with PSA recurrence > 3 years, Gleason < 7, less than pT3a, and PSA-DT > 12 months. These low-risk patients respond very well to salvage RT therapy, but this decision should be taken after considerations of pros and cons, and life expectancy. Patients within the high-risk group need early and aggressive salvage treatment.

Standard workup to detect metastases includes bone scan and abdominopelvic CT scan, but the diagnostic yield is poor on asymptomatic patients. They should only be considered in patients who have a higher than 10 ng/mL baseline PSA, high PSA kinetics (PSA-DT < 6 months), or symptomatic bone disease. As choline-PET is dependent of PSA values, it cannot be recommended if PSA < 1 ng/mL.

Therapeutic options for PSA-only rising recurrences are still controversial. They include: RT to the prostatic bed, continuous ADT, intermittent ADT, and observation. If primary treatment was RT, salvage RP, cryotherapy or brachytherapy may be indicated in selected patients without evidence of metastases and confirmed local recurrence, but they are still experimental [5, 6].

Early ADT shows no progression-free or disease-specific survival benefit compared with late ADT in two large comparative studies, although a favorable effect is observed in patients with shorter PSA-DT and/or high-risk tumor characteristics. If salvage ADT is considered, an intermittent strategy may be used as non-inferiority was demonstrated in a comparative trial in relation to continuous ADT in patients primarily treated with RT [7]. Nevertheless, patients with low-risk features (PSA-DT > 12 months, time to PSA elevation > 3 years, Gleason < 7 and stage < pT3a) or unfit patients with a life expectancy lower than 10 years, observation might be a viable therapeutic option, until the development of clinically evident metastatic disease.

#### Recommendations

In PSA recurrence M0 disease after RP, salvage RT is an option. Level of evidence: II. Strength of recommendation: A.

Standard work-up in this setting are bone scan if the patient have higher than 10 ng/mL baseline PSA or symptomatic bone disease, and choline-PET only if PSA ≥ 1 ng/mL. Level of evidence: III. Strength of recommendation: A.

If primary treatment was RT, salvage RP, cryotherapy or brachytherapy may be indicated in selected patients. Level of evidence: III. Strength of recommendation: B.

ADT or intermittent ADT are options in patients treated with RT. Level of evidence: I. Strength of recommendation: A. In unfit patients or with low-risk features, observation is an option. Level of evidence: III. Strength of recommendation: B.

Surgical castration, LHRH agonist or antagonist is standard of care for ADT. Level of evidence 1b. Strength of recommendation A.

### ADT-naïve M1 patient

ADT must be instituted in symptomatic patients, but there is some controversy about the best time to start ADT in asymptomatic patients. Review of good-quality trials showed no improvement in OS, although early ADT significantly reduce disease progression and its complications. ASCO guidelines concluded that it was not possible to make a recommendation on when to start ADT in advanced asymptomatic patients [8]. Anyway, deferred castration treatment in a well-informed patient needs close monitoring. In asymptomatic well-informed patients, intermittent ADT might be an option after an induction period. Some trials show the efficacy of this approach although the largest SWOG trial did not demonstrate non-inferiority against continuous ADT and patient should be informed. Non-steroidal antiandrogen monotherapy is less effective than castration in terms of OS, clinical progression and treatment failure and should not be offered to patients [9].

#### Recommendation

ADT must be instituted in symptomatic patients. Level of evidence: I. Strength of recommendation: A.

### Abiraterone in hormone-sensitive metastatic prostate cancer (mHSPC)

Recently, new data have been presented confirming the use of abiraterone in the context of advanced hormone-sensitive prostate cancer. The first phase III study (LATITUDE) is a double-blind trial in which 1199 patients who debuted with metastatic hormone-sensitive prostate cancer were randomized to treatment (ADT) with abiraterone (1000 mg/day) plus prednisone (5 mg/day) or placebo [10]. The main objectives were overall survival (OS) and radiological progression-free survival (SLPR). With a 30-month follow-up, the median OS was higher in the abiraterone group than in the placebo group: unreached versus 34.7 months (HR 0.62 [95% CI 0.51–0.76], *p* < 0.001). In addition, SLPR reached 33 months in the abiraterone group compared to 14.8 months in the placebo group (HR 0.47 [95% CI 0.39–0.55], *p* < 0.001). Abiraterone was significantly superior to placebo in other secondary endpoints: time to pain development, initiation of chemotherapy, progression of PSA, and the occurrence of a symptomatic bone event. The conclusion of the LATITUDE study is that the addition of abiraterone and prednisone to ADT in patients with newly diagnosed advanced hormone-sensitive prostate cancer increases OS and SLPR versus placebo.

In the second study (STAMPEDE), 1917 patients were included, 52% had metastatic disease, 20% were M0 with positive or indeterminate node and 28% M0N0 [11]. In 95% of the cases, the disease had been recently diagnosed. Patients were randomized to either ADT or ADT with abiraterone (1000 mg daily with 5 mg prednisolone; in the M0 setting, abiraterone was administered during 2 years). The main objective of the study was OS. Treatment was continued until radiological, clinical or biochemical progression. With a median follow-up of 40 months, 184 deaths were recorded in the abiraterone group versus 262 in the ADT arm (HR 0.63 [95% CI 0.52–0.76], *p* < 0.001). The HR was 0.75 for the M0 group and 0.61 for the M1. Authors concluded that in patients with locally advanced or metastatic prostate cancer, ADT in combination with abiraterone and prednisolone is associated with a significant increase in OS and PFS.

#### Recommendation

Abiraterone plus ADT is recommended in mHSPC patients.

Level of evidence: I. Strength of recommendation: A.

### Chemotherapy in hormone-sensitive metastatic prostate cancer (mHSPC)

Three phase III clinical studies have evaluated the role of docetaxel in this setting. The E3805 (CHAARTED) study was performed in 790 androgen sensitive metastatic prostate cancer patients who were randomized to receive continuous ADT alone or in combination with 6 cycles of docetaxel chemotherapy at standard dose (75 mg/m^2^). With a median follow-up of 28.9 months, the median OS was 57.6 months in the docetaxel plus ADT arm and 44.0 months in the ADT arm [HR 0.61; 95% CI (0.47, 0.80); *p* = 0.0003]. In patients with high-volume disease (defined as four or more bone metastases with at least one beyond the pelvis and vertebral column or visceral metastases), a difference of 17 months [HR 0.60; 95% CI (0.45, 0.81); *p* = 0.0006] was observed in the median OS with docetaxel plus ADT compared with ADT alone (49.2 vs. 32.2 months, respectively). In patients with low-volume disease, median OS had not been reached. In an updated analysis reported in the last ESMO meeting in 2016, with a median follow-up of 53.7 months, the clinical OS benefit of docetaxel plus ADT was significant only in high-volume disease [12]. However, in all secondary endpoints the benefit was observed in all subgroups.

The second trial, GETUG-AFU 15, randomized 192 patients to receive ADT plus docetaxel (up to nine cycles) or ADT alone. After a median follow-up of 50 months, median OS was 58.9 months in the ADT plus docetaxel arm and 54.2 months in the ADT arm [HR 1.01; 95% CI (0.75, 1.36); *p* = 0.955] [13].

The last phase III study is STAMPEDE trial, a multi-arm, multi-stage trial whose control arm is the standard ADT treatment. 2962 patients with high-risk, locally advanced, metastatic or recurrent prostate cancer were randomized to four groups in a 2:1:1:1 allocation to standard of care (ADT alone), standard of care plus zoledronic acid, standard of care plus docetaxel and standard of care plus zoledronic acid plus docetaxel. After a median follow-up of 43 months, OS in the ADT plus docetaxel arm was 81 months versus 71 months in the control arm [HR 0.78; 95% CI (0.66, 0.93); *p* = 0.005]. In the metastatic subgroup (61%), OS in the ADT plus docetaxel was 60 versus 45 months [HR 0.76; 95% CI (0.62, 0.92); *p* = 0.005] [14].

A systematic review and meta-analysis of the available randomized studies was performed, and these three trials of docetaxel in mHSCP were analyzed (CHAARTED, GETUG-15 and STAMPEDE), with a total of 2992 patients, obtaining a HR of 0.77 [95% CI 0.58, 0.70; *p* < 0.0001), with a 9% absolute improvement in survival at 4 years with ADT plus docetaxel relative to ADT alone [15].

#### Recommendation

The results of a meta-analysis with all published phase three trials allow to recommend the use of docetaxel plus ADT in mHSPC patients fit enough for chemotherapy. However, in some studies with a smaller sample size or lower follow-up, a benefit in OS in patients with low-volume disease has not been conclusively demonstrated.

Level of evidence: I. Strength of recommendation: A.

## Treatment of castration-resistant prostate cancer

### Definition of castration-resistant prostate cancer (CRPC)

CRPC is defined by disease progression despite ADT and may present as one or any combination of a continuous rise in serum levels of PSA, progression of pre-existing disease or appearance of new metastases.

In their second publication, the Prostate Cancer Clinical Trials Working Group 2 (PCWG2) defines patients with CRPC as patients with castrate serum levels of testosterone (testosterone < 50 ng/dL or 1.7 nmol/L) plus biochemical or radiological progression despite anti-androgen withdrawal for at least 4–6 weeks [16]. Biochemical progression is defined as three consecutive rises in PSA 1 week apart, resulting in two 25% increases over the nadir, and PSA > 2 ng/mL. Radiologic progression is defined when the appearance of new lesions: either two or more new bone lesions on bone scan or a soft tissue lesion using the Response Evaluation Criteria in Solid Tumors (RECIST).

PCWG2 advises investigators not to wait to assess for a withdrawal response in patients who did not respond or who showed a decline in PSA for 3 months or less after an antiandrogen was administered as a second-line or later intervention.

#### Continuing treatment with luteinizing hormone-releasing hormone analogs in patients with castration-resistant prostate cancer

When disease progresses, discontinuation LHRH analogs therapy can result in an increase in serum testosterone, and thus, contribute to disease progression. Continuation of treatment with LHRH analogs in patients with castration-resistant disease remains controversial, but exogenous testosterone has been demonstrated to exacerbate disease in the metastatic setting [17].

Two trials have shown a marginal survival benefit for patients with metastatic CRPC (mCRPC) remaining on LHRH analogs during second- and third-line therapies [18, 19]. In addition, all subsequent treatments have been studied in men with ongoing androgen suppression; therefore, it should be continued indefinitely in these patients.

#### Recommendation

- LHRH analogs should be continued in patients with CRPC. Level of evidence: III. Strength of recommendation: C.

### Asymptomatic or minimally symptomatic patients with mCRPC

To date, three randomized phase III trials have demonstrated increased survival in patients with asymptomatic or minimally symptomatic mCRPC. The three studies included patients with PS equal to 0–1, with a low level of pain as measured by the Brief Pain Inventory-Short Form Scale (BPI-SF) equal to 0–1 (asymptomatic) or 2–3 (minimally symptomatic), respectively. In these trials, metastatic disease was documented.

In 2010, the IMPACT study [20], sipuleucel-T, an autologous active cellular immunotherapy agent, prolonged OS among men with mCRPC, before or after docetaxel treatment, with a relative reduction of 22% in the risk of death as compared with the placebo group (hazard ratio [HR]: 0.78; 95% CI 0.61–0.98; *p* = 0.03). This reduction represented a 4.1 month improvement in OS (25.8 months vs. 21.7 months). The most common associated adverse events (AEs) were chills (51%), fever (22%), fatigue (16%), nausea (14%) and headache (11%). Sipuleucel-T is not available in Europe.

In the second study (COU-AA-302) [21], abiraterone in combination with prednisone was superior to placebo plus prednisone. Overall survival, radiographic progression-free survival (rPFS) and secondary endpoints all favored the abiraterone arm (in terms of time to initiation of cytotoxic chemotherapy, opiate use for cancer-related pain, PSA progression and decline in performance status). After a median follow-up of 22.2 months, there was significant improvement of rPFS (median: 16.5 months vs. 8.2 months; HR 0.52; *p* < 0.001), and the trial was unblinded. At the final analysis, with a median follow-up of 49.2 months, the OS endpoint was significantly positive (34.7 months vs. 30.3 months; HR 0.81; 95% CI 0.70–0.93; *p* = 0.0033) [7]. With regard to toxicity, adverse events related to mineralocorticoid excess and liver function abnormalities were more frequent with abiraterone but were mostly grades 1–2. The role of abiraterone in the groups of patients not included in the COU-AA-302 study, especially in patients with symptomatic or visceral metastases, is controversial.

PREVAIL was a phase III trial comparing enzalutamide activity with placebo in asymptomatic or minimally symptomatic chemotherapy-naïve mCRPC patients [22, 23]. Unlike the COU-AA-302 study, approximately 12% of the patients had visceral metastases (lung and/or liver). Enzalutamide demonstrated significant improvement in both co-primary end points of rPFS (HR 0.186; 95% CI 0.15–0.23; *p* < 0.0001) and OS (HR 0.706; 95% CI 0.6–0.84; *p* < 0.001). It also showed a benefit with respect to all secondary end points, including the time to initiation of chemotherapy, the time until first skeletal-related event (SRE), complete or partial soft tissue response, time to PSA progression and rate of decline of at least 50% in PSA. The most common clinically relevant adverse events were fatigue and hypertension.

A significant improvement in OS of 2.0–2.9 months occurred with docetaxel-based chemotherapy compared with mitoxantrone plus prednisone therapy [24]. No comparative studies have been conducted with docetaxel against new hormonal treatments. There are general factors that predict a rapidly progressive disease and shorter survival and could justify the choice of docetaxel as first line: the presence of anemia, multiple metastatic sites, elevated LDH and alkaline phosphatase, and a PSA-DT of less than 55 days [25, 26]. In these patients, docetaxel-based chemotherapy would be the treatment of choice.

#### Recommendations

- Sipuleucel-T would be a treatment option in asymptomatic patients with mCRPC if regulatory approval is obtained in Europe. Level of evidence: Ib. Strength of recommendation: A.
- Abiraterone is a treatment option for asymptomatic or minimally symptomatic patients with mCRPC without visceral metastases and previously untreated with chemotherapy. Level of evidence: Ib. Strength of recommendation: A.
- Enzalutamide is a treatment option for asymptomatic and minimally symptomatic patients with mCRPC, including selected patients with visceral metastases, who have not received previous chemotherapy. Level of evidence: Ib. Strength of recommendation: A.
- Patients with asymptomatic or minimally symptomatic mCRPC and adverse prognostic factors (the presence of visceral metastases) should also be considered for docetaxel treatment. Level of evidence: 1a. Strength of recommendation: A.

### First-line therapy for symptomatic mCRPC

In contrast to asymptomatic/mildly symptomatic disease, the definition of symptomatic mCRPC status needs further explanation. Noticeably, this definition requires of the presence of symptoms clearly attributable to disease burden, which frequently in mCRPC are identified as pain. Various contemporaneous trials have defined symptomatic patients as those who present with a BPI-SF score > 3 and/or regular opiates for pain control [20–22]. Nonetheless, the definition of a symptomatic mCRPC patient in a daily clinical practice should warrant further assessment than just a pain scale. For example, many patients reported symptoms different from pain but clearly related to metastases and tumor burden in a recent large survey conducted by the International Prostate Cancer Coalition (results available at MenWhoSpeakUp.com).

Since 2004, docetaxel has become the preferred cytotoxic chemotherapy option for symptomatic mCRPC. Two phase III studies, TAX327 [24] and SWOG99-16 [27] demonstrated the superiority of docetaxel (± estramustine) over the mitoxantrone plus prednisone. The TAX327 (*n* = 1006) patients compared (a) 3-week docetaxel 75 mg/m^2^, (b) 1-week docetaxel 30 mg/m^2^, and (c) 3-week mitoxantrone 12 mg/m^2^; all patients received continuous oral prednisone 5 mg bid. The 3-week docetaxel arm was superior to the mitoxantrone arm with a OS of 18.9 months versus 16.5 months, respectively (HR 0.76, *p* = 0.009), and has been confirmed (19.2 vs. 16.3) in a later updated analysis with 867 deaths [28]. Bone pain responses were more significant in docetaxel patients (35% vs. 22%; *p* = 0.08), as were improvements in quality of life (QoL) compared to the mitoxantrone group. The weekly docetaxel arm was not significantly superior to the mitoxantrone arm in terms of OS, pain reduction or improvement of QoL [24]. The SWOG99-16 also supported the superiority of docetaxel plus estramustine over mitoxantrone in terms of OS but with a significant increase in toxicity in the experimental arm [27]. A phase III study by Kellokumpu-Lehtinen et al., showed a significantly lower rate of grades 3–4 AEs with 2-week docetaxel at 50 mg/m^2^ compared with 3-week docetaxel [29]. Although the use of the 2-week schedule has been proposed as an alternative for unfit patients, it should be cautiously considered. First, this study did not meet the predesigned primary endpoint, and second, data on comorbidities were not reported and only 6% of patients included had poor performance status (PS 2).

The Firstana Study (NCT01308567), which was presented at ASCO 2016 [30] but has not been published yet, compared 3-week cabazitaxel 25 mg/m^2^ (C25) to 3-week cabazitaxel 20 mg/m^2^ (C20) and 3-week docetaxel 75 mg/m^2^ (D) all in combination with prednisone 5 mg bid as first-line chemotherapy in men with mCRPC, has failed to show superiority of cabazitaxel in median OS (C25 25.2 vs. C20 24.5 vs. D 24.3 months). However, toxicity profiles for cabazitaxel and docetaxel differ. Febrile neutropenia, diarrhea, and haematuria occurred more often with C25 in while peripheral neuropathy, oedema, alopecia, and nail disorders were associated with docetaxel.

The ALSYMPCA study randomized mCRPC patients with symptomatic bone metastases and no known visceral metastatic disease to receive six doses of Ra-223 every 4 weeks at 50 kBq/kg (55 kBq/kg following 2015 NIST update) versus best supportive care (BSC). In this study, definition of asymptomatic patient was closer than in COU-AA-302. The study showed a significant improvement in median OS with Ra-223 (14.9 vs. 11.3; *p* < 0.001). Ra-223 was also associated with longer time to first SRE, improved pain, and improved QoL [31]. This study included patients unfit for docetaxel or unwilling to have chemotherapy, and then Ra-223 could be indicated in patients either pre- and post-docetaxel without an apparent detriment in benefit from Ra-223 or the subsequent chemotherapy lines [32].

Although abiraterone plus prednisone or enzalutamide has not been studied as first-line treatment for symptomatic mCRPC patients, they both have shown activity in a symptomatic population after docetaxel with a favorable safety profile. The APCCC 2015 [33] supported to extrapolate the data of the COU-AA-301 [34] and AFFIRM [35] study to the first-line symptomatic mCPRC population. Then they could be considered as alternative for those patients rejecting chemotherapy or unfit for such treatment.

The incorporation of docetaxel to the castration-naive metastatic (mCNPC) setting has generated new questions about sequencing other therapies with regards to the first-line in mCRPC. When there are not specific studies analyzing the best therapy in this setting, similar approaches to patient who undergo a second-line treatment after docetaxel may be considered.

#### Recommendations

- Docetaxel at 75 mg/m^2^ every 21 days plus prednisone 5 mg bid is the preferred first-line treatment for symptomatic mCRPC naïve for docetaxel. Level of evidence: I. Strength of recommendation: A.
- Docetaxel at 50 mg/m^2^ every 15 days may be considered as potentially less toxic alternative. Level of evidence: II. Strength of recommendation: C
- Ra-223 at 55 mBq/kg every 28 days for six cycles could be offered to mCRPC patients naïve for docetaxel, with symptomatic bone metastases and not known visceral metastases how are unfit or are not willing to receive docetaxel (Level of evidence: I. Strength of recommendation: A)
- Abiraterone or enzalutamide could be considered as an alternative first-line treatment for symptomatic mCRPC patients naïve for docetaxel unfit or unwilling to receive docetaxel. Level of evidence: V. Strength of recommendation: A

### Second-line therapy: cabazitaxel and second-line hormone therapy

Cabazitaxel and hormone therapies, enzalutamide and abiraterone have been tested in large randomized phase III trials in mCPC patients that progressed to docetaxel [34–36].

*Cabazitaxel* is a semisynthetic taxane that acts promoting tubulin assembly and stabilizing microtubules that was selected for clinical development based on a better anti-proliferative activity than docetaxel against chemotherapy-resistant tumor cell lines. It is approved for the treatment of patients with mCRPC who had progressed to docetaxel, after the results of the TROPIC trial [36].

The TROPIC trial was an open-label randomized phase III trial in men with metastatic CRPC patients who had received previously docetaxel. Cabazitaxel (25 mg/m^2^ every 3 weeks) prednisone was compared with mitoxantrone 12 mg/m^2^, up to 10 cycles, both in combination with continuous prednisone (10 mg/day PO). A total of 755 patients were included. The primary endpoint was OS, which was 15.1 months in the cabazitaxel and 12.7 months in the mitoxantrone group (HR was 0.70 (95% CI 0.59–0.83, *p* < 0.0001). Secondary endpoints included PFS and safety. Median PFS was 2.8 months in the cabazitaxel arm and 1.4 months in the mitoxantrone arm HR 0.74, 0.64–0.86, *p* < 0.0001). Cabazitaxel also showed significant improvement in PSA and objective response rate and time to PSA progression. Updated data with a median follow-up of 25.5 months showed that more patients remained alive at 2 years following cabazitaxel than mitoxantrone (odds ratio, 2.11; 95% CI 1.33–3.33) [37]. Cabazitaxel showed more toxicity than mitoxantrone, being neutropenia and diarrhea the most common clinically significant grade 3 or higher toxicities that occurred in 82 and 58% of patients treated with cabazitaxel. Moreover, 28 (8%) patients in the cabazitaxel group had febrile neutropenia respect to only 5 (1%) in the mitoxantrone arm [36]. Thus, granulocyte colony-stimulating factor administered prophylactically in the high-risk patient population is recommended.

To study if lower doses of cabazitaxel may induce similar antitumor activity with lower toxicity, the Phase III trial PROSELICA was designed. The main objective was to demonstrate the non-inferiority in OS of the dose of 20 mg/m^2^ respect to 25 mg/m^2^ [38] 1200 patients were included, showing that 20 mg/m^2^ was not inferior in OS and had a lower degree of grade III–IV toxicity. However, the PSA- and objective response rates were superior for the higher dose, 42.5% versus 29% and 23.4% versus 18.5%, respectively. Standard recommended dose for cabazitaxel is 20 mg/m^2^.

*Hormone-therapy* Two phase III trials established the role of abiraterone and enzalutamide in mCRPC progressing after docetaxel. The mechanism of action for both drugs has been described in the “first line” section.

The COU-A-301 trial randomized in a 2:1 ratio, 1195 patients to receive 1000 mg of abiraterone acetate (797 patients) or placebo (398 abiraterone), both plus continuous prednisone 10 mg/daily [34]. Overall survival was longer in the abiraterone group (14.8 months vs. 10.9 months; hazard ratio, 0.65; *p* < 0.001). Abiraterone was also superior to placebo in all secondary end points, including time to PSA progression (10.2 months vs. 6.6 months; *p* < 0.001), PFS (5.6 months vs. 3.6 months; *p* < 0.001), and PSA response rate (29% vs. 6%, *p* < 0.001). The most common adverse events for abiraterone were fluid retention, hypertension, and hypokalemia. This benefit for abiraterone was maintained in an updated follow-up publication (median follow-up of 20.2 months) [39]. Moreover, it improves the quality of life, the pain control and delay and reduced the risk of bone-related events [39, 40].

The AFFIRM study was a phase 3, double-blind, placebo-controlled trial, that randomized 1199 patients (2:1 ratio), to receive enzalutamide, 160 mg per day (800 patients) or placebo (399 patients) [35]. Primary endpoint was OS. The median OS was 18.4 months in the enzalutamide group versus 13.6 months in the placebo group (HR 0.63; *p* < 0.001). The superiority of enzalutamide over placebo was also observed with respect to all secondary endpoints: PSA response rate (54% vs. 2%, *p* < 0.001), objective response rate (29% vs. 4%, *p* < 0.001), the quality-of-life response rate (43% vs. 18%, *p* < 0.001), the time to PSA progression (8.3 months vs. 3.0 months; hazard ratio, 0.25; *p* < 0.001), radiographic PFS (8.3 months vs. 2.9 months; hazard ratio, 0.40; *p* < 0.001), and the time to the first SRE (16.7 months vs. 13.3 months; hazard ratio, 0.69; *p* < 0.001) [41]. Rates of fatigue, diarrhea, and hot flashes were higher in the enzalutamide group. Seizures were reported in five patients (0.6%) receiving enzalutamide.

Abiraterone has minimal activity after enzalutamide treatment; in a phase IV trial, PFS was 5.8 months and only 2% of patients had a PSA decline of greater than 50%. On the other hand, enzalutamide after abiraterone treatment show some activity, with a 50% of PSA response in 22 of 33 patients of the post hoc analysis of COU-AA 302 trial [42, 43]; although in the only prospective phase II trial that compares enzalutamide in patients with progressive mCRPC after ≥ 24 weeks of abiraterone acetate plus prednisone treatment, rPFS was 8 months [44].

#### Recommendation

There are several options of choice after docetaxel treatment, according to patient characteristics and therapy received prior to docetaxel (today, most patients receive abiraterone or enzalutamide as a first-line treatment of CPCR)

##### Cabazitaxel

Level of evidence: I. Strength of recommendation: A

##### Abiraterone

In patients without prior enzalutamide. Level of evidence: I. Strength of recommendation: A

In patients with prior enzalutamide it may be considered in selected cases. Level of evidence: IV. Strength of recommendation: D

##### Enzalutamide

In patients without prior abiraterone. Level of evidence: I. Strength of recommendation: A

In patients with prior abiraterone, it may be considered in selected cases. Level of evidence: IV. Strength of recommendation: D

## Treatment of bone disease

Bone metastases are present in more than 90% of patients with CRPC and are associated with increased morbidity, mortality and cost due to bone events. The recent PCWG3 criteria recommends the use for clinical trials of the term “symptomatic skeletal events” (SSEs), defined as symptomatic fracture, need of surgery or radiation to bone (due to pain or other causes), or spinal cord compression [45]. The classical term SRE, also included asymptomatic nonclinical fractures and even malignant hypercalcemia. Oncologists should be aware of preventing and treating these complications.

Besides analgesics and the appropriate systemic oncologic therapy, local palliative RT should be always considered to treat oncologic pain due to localized growth of bone metastases. Spinal cord compression (5–10% of cancer patients) is an oncologic emergency requiring early diagnosis and immediate treatment. Patients should be started on dexamethasone (16 mg/day). Decompressive surgery followed by long-course RT can be offered to patients with spinal instability, displacement of vertebral fragment, a life expectancy of at least 3–6 months and those without histopathologic diagnosis. Otherwise, palliative radiotherapy is indicate [46].

Surgery is also indicated in patients with metastases in long bones (femur, peri-acetabular region or humerus) when there is a reasonable risk of fracture, as well as in cases of spinal instability. Less invasive procedures such as vertebroplasty (percutaneous injection of cement, usually methyl methacrylate) into the vertebral body, or kyphoplasty (insertion of a balloon into the fractured vertebral body) are indicated in selected cases for relieving pain, preventing vertebral fracture, and restoring the height of the vertebral body produced after vertebral collapse [47].

The inhibition of osteoclasts with the bisphosphonate zoledronic acid (ZA), or the humanized monoclonal antibody (IgG2) to RANK ligand denosumab (DNS) are effective systemic therapies in preventing and delaying cancer-related skeletal events. ZA is currently indicated for the prevention of SRE in adult patients with mCRPC and bone metastases, or with tumor-induced hypercalcaemia [48]. ZA is not indicated in non-CRPC [15]. The recommended dose is 4 mg administered as an intravenous infusion of at least 15 min, every 3 or 4 weeks for a maximum of 2 years. Up to 3% of cases of altered renal function have been described in relation to ZA that may be increased in patients with dehydration, pre-existing renal failure, or nephrotoxic drugs. Doses of ZA should be reduced in patients with creatinine clearance (ClCr) < 60 mL/min, and avoided in cases of ClCr < 30 mL/min.

Denosumab is indicated for the prevention of SREs in adults with bone metastases of solid tumors. The recommended dose is 120 mg given as a single injection subcutaneously every 4 weeks. No dose adjustment is required in patients with renal insufficiency. Experience in patients undergoing dialysis or with severe renal insufficiency (ClCr < 30 mL/min) is limited. In a randomized study including 1904 patients with CRPC, DNS was better than zoledronic acid for delaying of SREs. The proportion of SRE was 36% versus 41%. Median time to first on-study SRE was 20.7 months (95% CI 18.8–24.9) with DNS versus 17.1 months (15.0–19.4) with ZA (HR 0.82, 95% CI 0.71–0.95; *p* = 0.0002 for non-inferiority, and *p* = 0.008 for superiority). Overall survival and investigator-reported disease progression were not significantly different between both groups. More events of hypocalcaemia occurred in the DNS arm (13% vs. 6%, *p* < 0·0001). Osteonecrosis of the jaw was infrequent (2% vs. 1%), *p* = 0.09). Acute phase reactions due to drug administration occurred in 8% of patients on DNS and 18% on ZA, and adverse events potentially associated with renal impairment, in 15% vs. 16%, respectively [49].

All patients receiving ZA or DNS should take supplements of at least 500 mg of calcium and 400 IU of vitamin D daily unless hypercalcemia is present.

### Recommendations

- Zoledronic acid and denosumab are effective systemic therapies in preventing and delaying cancer-related skeletal events in CRPC. Level of evidence: I. Strength of recommendation: A
- Zoledronic acid is not indicated in non-CRPC. Level of evidence: I. Strength of recommendation: A
- Denosumab is superior to zoledronic acid for delaying skeletal-related events in CRPC. Level of evidence: I. Strength of recommendation: A

## Radium 223

Radium-223 is the first therapy directed against the bone microenvironment and the first form of radiation therapy to show antitumor efficacy. The main difference with other therapeutic radioisotopes capable to accumulate in bone turnover sites is that it is an alpha emitter. In comparison to beta-emitting radioisotopes, strontium-89 and samarium-153, used in the palliation of metastatic bone pain, alpha emitters deliver radiation with a higher biological effect to a more localized area (a range of 2–10 cell diameters), causing high double-strand DNA breaks leading to cell death [50].

In the ALSYMPCA trial [31], eligible patients were those with mCRCP with symptomatic bone metastases. Bone metastases were identified by the presence of two or more bone metastases in the bone scan, and symptomatic disease was described as the regular use of any analgesic medication or treatment with external-beam radiation therapy. Patients were excluded if they had received chemotherapy within the previous 4 weeks or had not recovered from adverse events due to chemotherapy. Additional exclusion criteria were previous hemi-body external radiotherapy, systemic radiotherapy with radioisotopes within the previous 24 weeks, a blood transfusion or use of erythropoietin-stimulating agents within the previous 4 weeks, a malignant lymphadenopathy that was more than 3 cm in the short-axis diameter, a history of or the presence of visceral metastases, and imminent or established spinal cord compression.

In this study, patients were randomized 2–1 to receive either radium 223 or placebo for a 6-month treatment period. Treatment was administered every 4 weeks for a total of six administrations at doses of 50 kBq/kg (55 kBq/kg following 2015 NIST update). Patients could receive also the best treatment for their situation with the exception of chemotherapy; these treatments may include radiotherapy (as previous mentioned), antiandrogens, corticoids, ketoconazole, estrogens and other treatments. The use of these different treatments was considered the best standard of care.

The main objective of the study was OS. This objective was reached, median OS (in the update analysis) for those patients who received active treatment was 14.9 months versus 11.3 months in the control arm (hazard ratio [HR] 0.70; 95% CI 0.55–0.88; *p* < 0001). All main secondary efficacy end points provided support for the benefit of radium-223 plus the best standard of care over placebo: radium-223, as compared with placebo, significantly prolonged the time to the first symptomatic skeletal event (median, 15.6 months vs. 9.8 months; hazard ratio, 0.66; 95% CI 0.52–0.83; *p* < 0.001), the time to an increase in the total alkaline phosphatase level (hazard ratio, 0.17; 95% CI 0.13–0.22; *p* < 0.001) and the time to an increase in the PSA level (hazard ratio, 0.64; 95% CI 0.54–0.77; *p* < 0.001).

This study also showed to have a favorable toxicity profile with most treatment-related adverse events being generally mild and manageable; the number of patients who developed adverse events (AE) was consistently lower in the Ra-223 group than in the placebo group for all AE (93% vs. 96%) as well as for grade 3/4 AEs (56% vs. 62%) [12]. Similarly, fewer patients on Ra-223 than on placebo had to stop therapy due to AEs (16% vs. 21%). Nonetheless, there are specific Ra-223-related toxicities that are frequent and should be monitored during treatment. A low incidence of myelosuppression was observed with most patients developing only grade 1/2 severity, such as anemia in 18.5% of patients, thrombocytopenia in 5% of patients, and neutropenia in 2.8%. Severe hematologic toxicity was infrequent and similar between Ra-223 and placebo groups, with grade 3/4 anemia in 12.6 and 13.2%, neutropenia in 2.1 and 0.7%, and thrombocytopenia in 6.5 and 2% of patients with Ra-223 and placebo, respectively. Moreover, grade 3/4 febrile neutropenia was extremely rare, occurring only in < 1% of patients. Final safety data from the 3-year long-term follow-up of the ALSYMPCA trial confirmed a similar good tolerability of Ra-223 with slightly more patients developing neutropenia (all grades, 5% vs. 1%) and thrombocytopenia (all grades, 12% vs. 6%) with Ra-223 than with placebo.

### Recommendation

The results of a phase III study demonstrate that radium 223 could be a useful drug in patients who have symptomatic bone metastases previously or not treated with docetaxel and without visceral metastases.

Level of evidence: I. Strength of recommendation: A.

## Personalized medicine in advanced prostate cancer

The molecular etiology of the resistance to treatment in prostate cancer is poorly understood, with studies being hindered by poor preclinical models and difficulty to acquiring metastatic tissue in advanced castration-naive and resistant prostate cancer. Then, we still treat all them as one disease and despite high intra- and inter-patient biological heterogeneity; currently treatment decisions are largely based in simple clinical or analytical variables but the analytical qualification and clinical validation of molecular stratification biomarkers to predict treatment benefit are urgently needed. Some of the most promising biomarkers include tissue-based or blood-borne markers such as *TMPRSS2*-*ERG*, *PTEN*, DNA damage response (DDR) defects, AR amplification, mutations or splicing.

The role of *TMPRSS2*-*ERG* gene fusion as a prognostic and potential predictive biomarker has been investigated in multiple settings. In example, Attard et al. [51] have demonstrated that patients with *TMPRSS2*-*ERG* with amplification of *ERG* derived the greatest benefit when treated with abiraterone and prednisone. On the other hand, Reig et al. [52] have associated *TMPRSS2*-*ERG* expression with poor outcomes in taxanes-treated mCRPC. Besides, Ferraldeschi et al. [53] associated pTEN loss with worse prognosis in mCRPC patients treated with abiraterone, and more recently de Bono et al. [54] have shown in randomised Phase II trial that the addition of ipatasertib (an AKT inhibitor) to abiraterone plus prednisone could improve outcomes in patients with *PTEN* loss. The IPATential150 phase III study will address the utility of adding ipatasertib to abiraterone and prednisone in *PTEN* loss patients (NCT03072238). The role of *RB1* loss, *Myc* amplification [55, 56] in relation to treatment response and to a potential neuroendocrine trans-differentiation will also be addressed in an increasing number of studies.

In the recent years, germ line or somatic aberrations in DDR genes have been identified in up to 23% of mCRPC [57]. Up to 12% of these cases may involve germ line aberrations, which would also have a potential impact in patients’ relatives [58]. When the management of metastatic disease with DDR defects and the benefit derived from conventional treatments needs to be clarified, this population may benefit from using novel PARPi [59] and platin salts [60, 61]. The TOPARP phase II study suggested that a panel of mutation largely based in homologous recombination/Fanconi anemia pathway (i.e. *BRCA1* and *BRCA2*) and *ATM* aberrations has a sensitivity of 87.5% and a specificity of 93.9% to predict responses to olaparib [57]. These has lead to several phase II and III trials with olaparib (Profound study NCT02987543), niraparib (Galahad study NCT02854436), rucaparib (TRITON2 NCT02952534 and TRITON3 NCT02975934 studies) or talazoparib (NCT03148795) that will explore the utility of PARPi in patients with different DDR defects. The Swiss PRO-PLAT phase II trial (NCT02311764) is addressing the role of DDR in predicting response to carboplatin in mCRPC.

Finally, the detection of alternative splicing of *AR* such as *AR*-*V7* or *AR* aberrations has also shown some promise in predicting treatment benefit in mCRPC. Detection of *AR*-*V7* in EPCAM/HER2 positive circulating tumor cells (CTCs) by PCR has been linked to poor response rate and poor outcomes in patients treated with abiraterone or enzalutamide [62, 63] but may not have an impact when patients were treated with taxanes [64]. The ARMOR3 phase III trial in men expressing AR-V7 in CTCs compared enzalutamide with galeterone, a novel CYP17/AR inhibitor with activity against splicing variants. This study presented at ASCO 2017 was early terminated due to lack of efficacy in the experimental arm; still, a 42% of randomised patients to the enzalutamide arm responded with PSA declines > 50% [65]. Recently, Scher et al. have suggested that nuclear-specific AR-V7 protein localization is essential to guide treatment decisions [66, 67]. In addition, AR amplification and AR specific mutations in circulating tumor DNA have also shown some promise for future treatment stratification.
